# How attitudes generated by humanoid robots shape human brain activity

**DOI:** 10.1038/s41598-020-73728-3

**Published:** 2020-10-09

**Authors:** G. Di Cesare, F. Vannucci, F. Rea, A. Sciutti, G. Sandini

**Affiliations:** 1 Robotics, Brain and Cognitive Sciences Unit (RBCS), Istituto Italiano Di Tecnologia (IIT), Genoa, Italy; 2 Neuroscience Unit, Department of Medicine and Surgery, University of Parma, Parma, Italy; 3 Cognitive Architecture for Collaborative Technologies Unit (CONTACT), Istituto Italiano Di Tecnologia (IIT), Genoa, Italy

**Keywords:** Perception, Neuroscience, Social behaviour

## Abstract

During interpersonal interactions, people perform actions with different forms of vitality, communicating their positive or negative attitude toward others. For example, a handshake can be “soft” or “vigorous”, a caress can be ‘kind’ or ‘rushed’. While previous studies have shown that the dorso-central insula is a key area for the processing of human vitality forms, there is no information on the perception of vitality forms generated by a humanoid robot. In this study, two fMRI experiments were conducted in order to investigate whether and how the observation of actions generated by a humanoid robot (iCub) with low and fast velocities (Study 1) or replicating gentle and rude human forms (Study 2) may convey vitality forms eliciting the activation of the dorso-central insula. These studies showed that the observation of robotic actions, generated with low and high velocities, resulted in activation of the parieto-frontal circuit typically involved in the recognition and the execution of human actions but not of the insula (Study 1). Most interestingly, the observation of robotic actions, generated by replicating gentle and rude human vitality forms, produced a BOLD signal increase in the dorso-central insula (Study 2). In conclusion, these data highlight the selective role of dorso-central insula in the processing of vitality forms opening future perspectives on the perception and understanding of actions performed by humanoid robots.

## Introduction

During social interactions, people perform actions gently, neutrally, or rudely etc., expressing their positive or negative attitudes towards others. These aspects of actions characterize human behavior and provide information about the affective state of the agent. For example, when observing one individual greeting another, it may be immediately apparent if that person is happy or not, or if he/she feels good. The same observations occur within speech: when answering the phone, it is possible to ascertain how the other person feels from their tone of voice. These forms of communication have been named ‘vitality forms’ by Daniel Stern^1,2^.

It is important to note that vitality forms differ from emotions, especially from basic emotions, in several aspects: basic emotions are short-lasting events characterized by viscero-motor responses and preparation to act. According to Darwin^3^ and James^4^, these two characteristics represent the essence of emotional states. In contrast, vitality forms represent the way *(the form)* in which actions are performed and reflect the internal states of the agent; they modulate human behavior in a *continuous* manner.

Besides the goal (what) and motor intention (why), vitality forms represent a third fundamental aspect of the action, conveying information via the way in which actions are performed (how). Vitality forms play a double role in interpersonal interactions: the expression of vitality forms allows the agent to communicate his attitude, while the perception of vitality forms allows the receiver to understand the attitude of others. The ability to perceive and express vitality forms is already present in babies during mother–child interactions^5^, evidencing the important role that vitality forms play in relating to and understanding others. Perception of vitality forms is impaired in individuals with social and communicative disorders such as children with Autism Spectrum Disorders^6–8^. Most importantly, recent findings have shown that gentle and rude vitality forms expressed by the agent through voice or gesture, influence the subsequent motor response of the receiver, stressing even more their relevance in social interactions^9^. Despite the crucial role of vitality forms in human relations very little is known about these forms of social communication. To date, only Di Cesare and colleagues have investigated the neural correlates involved in vitality processing showing that the perception and expression of vitality forms result in activation of the dorso-central insula^10,11^. In a subsequent study, the same authors demonstrated that the same insular sector is activated, not only when participants *observed* or *imagined* performing hand actions with a specific vitality form, but also when they *listened to action verbs* or *imagined pronouncing* them gently or rudely^12^. Taken together, these findings clearly indicate that the dorso-central insula is a selective area involved in the encoding of vitality forms regardless of the modality through which they are conveyed^13^.

Besides obtaining a better understanding of this important social sign, the study of vitality forms may have important future impact. In the coming years, robots will be used in numerous different social contexts such as hospitals, education^14,15^, security, and health care^16^, interacting more and more with humans. The expression of vitality forms through action and speech could represent one of the missing links in the human–robot relationship that can allow a greater and immediate understanding of the robot’s mind and intentions^17,18^. The ability to express vitality forms represents a fundamental component a more human like robot relationship^17–20^. A fascinating possibility suggests that new generations of robots could be endowed with the capacity to express vitality forms, appearing more authentic and comfortable interacting by exploiting this intuitive social skill. An interesting question is whether the observation of actions generated by a humanoid robot (iCub) can convey vitality forms, and which kinematic parameters are important in eliciting the activation of the dorso-central insula.

In contrast with previous fMRI studies, which have investigated the understanding of robotic actions (what)^21,22^, the present study is focused on a different action property: the form (how). For this purpose two fMRI studies were conducted: the aim of the first study was to assess whether the observation of the iCub robot, generating actions with different velocity profiles, produced an activation of the insula. It has been shown that human actions performed with rude vitality forms are characterized by a higher velocity profile than those performed with gentle vitality forms. This stresses the important role of kinematic features and, in particular, velocity profile and peak velocity in conveying vitality forms^23^. In this study, sixteen participants were required to pay attention to either video-clips showing a human actor offering an object in a gentle or rude way, or to video-clips showing the iCub robot generating the same action with a low and high velocity. It is important to note that these robotic actions were performed with a “biological motion” signature (i.e. were in accordance with the 2/3 power law which characterizes biological motion)^40^. Results of this first study showed that the observation of human actions produced activation of the dorso-central insula whereas the observation of robotic actions performed with biological motion did not elicit insular activity. In other words this first experiment showed that modulating the velocity profile of robot actions according to the 2/3 power law does not necessarily produce vitality forms because the observation of these actions did not activate the insula.

In order to assess whether the lack of the insular activity was due to the presence of kinematics differences between human and robotic actions, or to the nature of the agent (human vs. robot), a second study was conducted: an actress was asked to perform an offering gesture gently or rudely and its kinematic parameters (i.e. peak velocity, length trajectory) were recorded by the Optotrack motion caption system and remapped into the kinematic model of the iCub robot allowing it to exactly replicate the human action including the parameters encoding the same vitality forms. It was then assessed whether the observation of these robotic actions, replicating the human kinematics, produced activation of the dorso-central insula. The results of this second study showed that the observation of robotic actions endowed with human vitality forms produced a BOLD signal increase in the dorso-central insula. This insular activity was very similar to that obtained during the observation of human actions.

In conclusion, the results obtained in these two fMRI studies provide evidence that the kinematic parameters of human actions represent an important cue essential to trigger the activity of the dorso-central insula. Furthermore it demonstrated that vitality forms are encoded by kinematic parameters (e.g. peak velocity) which are not simply related to modulating the velocity profile of biological motions compliant to 2/3 power law. These findings may have implications in neuroscience but also in other fields, such as robotics and, more generally, human–machine interaction suggesting that future humanoid robots and virtual avatars could be endowed with the capacity to generate vitality forms in order to promote human–robot interactions.

## Results

### fMRI study 1

Sixteen healthy right-handed volunteers (6 females and 10 males, mean age = 26, SD = 3) participated in the first fMRI study. Two conditions were presented: *human* and *robot*. In the human condition participants observed video-clips showing an actor performing an action (offering one of five possible objects) in a gentle or rude way (*human vitality forms*) or without any vitality form (control: *human* jerky action) (Fig. 3A). Given that gentle and rude human actions are characterized by different kinematic properties (velocity, trajectory), an attempt was made to obtain the same actions with iCub robot by manipulating the velocity of movements generated according to the 2/3 power law which characterizes biological motion. In other words, iCub robot was programmed to generate actions with high and low velocities. Thus, in the robot condition, participants were presented with video-clips showing the iCub robot performing a passing action with two velocities (low and high) (*robotic vitality forms*) or with a jerky movement (control: *robotic* jerky actions) (Fig. 3B). During the presentation of these stimuli, participants were asked to fixate on a white cross in the center of the screen and to focus on the way in which actions were performed.

#### Main effect of the observation of actions performed by human and iCub robot

**Figure 1:**
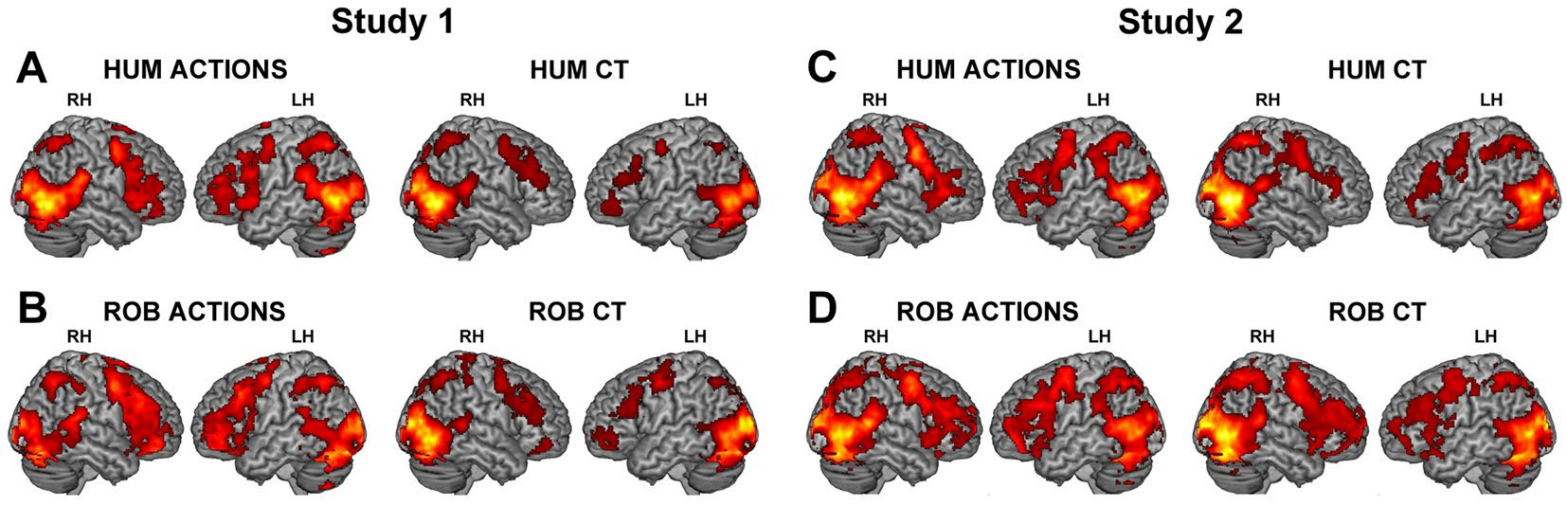
Brain activations resulting from the observation of gentle and rude human action vitality forms (global null conjunction) (**A**–**C**, left panel), robotic actions (**B**–**D**, left panel) and relative controls (jerky actions: human control, (**A**–**C**), right panel; robot control, (**B**–**D**), right panel) obtained in Study 1 and Study 2. These activations have been rendered into a standard Montreal Neurological Institute brain template (P_FWE_ < 0.05 cluster level). *LH* left hemisphere, *RH* right hemisphere.

The observation of human actions conveying gentle and rude vitality forms when compared with a baseline, resulted in activation of the occipital lobe, bilateral inferior parietal lobule extending to the supramarginal gyrus, and bilateral premotor cortex extending to the inferior frontal gyrus. In addition, there was a bilateral activation of the insular cortex. The results of the global null conjunction produced following observation of gentle and rude vitality forms are shown in Fig. 1A (left panel). A similar activation pattern of the parietal and frontal areas was observed for the robot condition with a large extension of the frontal areas (Fig. 1B, left panel). It is important to note the lack of the activity of the insular cortex (Fig. 1B, left panel; for coordinates see Table 1). In contrast, the observation of videoclips reproducing jerky actions performed by both human and iCub robot (control stimuli) produced the activation of the parieto-frontal circuit typically involved in the recognition and execution of actions but not of the insula (Fig. 1A,B, right panels).

**Table 1:** Cerebral activity obtained in Study 1 during the observation of (**A**) *human actions versus baseline* (global null conjunction of *gentle* and *rude* conditions); (**B**) *robotic actions versus baseline*; (**C**) *human actions versus robotic actions*. Local maxima, as shown in Fig. 1A,B, are given in MNI standard brain coordinates, significant threshold is set at *P*_FWE_ < 0.05 (cluster-level).

Anatomical region	Left hemisphere				Right hemisphere			
	x	y	z	Z-score	x	y	z	Z-score
**(A) Human actions versus baseline**								
Inferior occipital gyrus	− 6	− 78	− 10	Inf	4	− 82	− 2	Inf
Middle occipital gyrus	− 46	− 72	4	Inf	46	− 76	4	Inf
Lingual gyrus	− 6	− 78	− 10	Inf	4	− 82	− 2	Inf
Inferior temporal gyrus					50	− 68	− 8	Inf
Middle temporal gyrus					48	4	52	6.14
Inferior parietal lobule	− 28	− 52	42	Inf				
Precentral gyrus	− 36	− 4	50	5.87	38	0	46	7.62
Inferior frontal gyrus	− 38	24	− 4	5.79				
Posterior-medial frontal gyrus					− 10	0	68	5.67
Insula	− 44	12	− 2	4.97				
Hippocampus	− 26	− 22	− 10	5.35				
Thalamus	− 18	− 30	0	7.45				
**(B) Robotic actions versus baseline**								
Superior Occipital Gyrus	− 14	− 96	8	Inf				
Fusiform Gyrus	− 34	− 72	− 12	Inf	28	− 76	− 14	Inf
Lingual Gyrus					4	− 84	− 6	Inf
Calcarine Gyrus	− 2	− 84	− 12	Inf	16	− 94	2	Inf
Inferior Parietal Lobule	− 32	− 57	40	6.91	40	− 54	44	5.86
Angular Gyrus					38	− 58	52	6.00
Precentral Gyrus	− 40	− 4	52	6.62	40	2	48	7.50
Inferior Frontal Gyrus	− 36	50	− 16	6.41				
Middle Frontal Gyrus	− 46	24	40	7.15				
Orbital Gyrus					40	46	− 18	7.14
Middle Orbital Gyrus					48	48	− 8	6.41
Hippocampus	− 24	− 26	− 8	7.09	24	− 30	− 4	7.04
Thalamus	− 20	− 30	4	7.72				
Cerebellum	− 12	− 84	− 18	Inf	28	− 74	− 20	Inf
**(c) Human actions versus robotic actions**								
Lingual gyrus					6	− 66	2	6.52
Middle Temporal Gyrus	− 44	− 64	8	3.67				
Middle Frontal Gyrus	− 32	36	32	4.33				
Insula	− 38	8	10	3.96				

#### Contrasts between vitality forms and control stimuli

**Figure 2:**
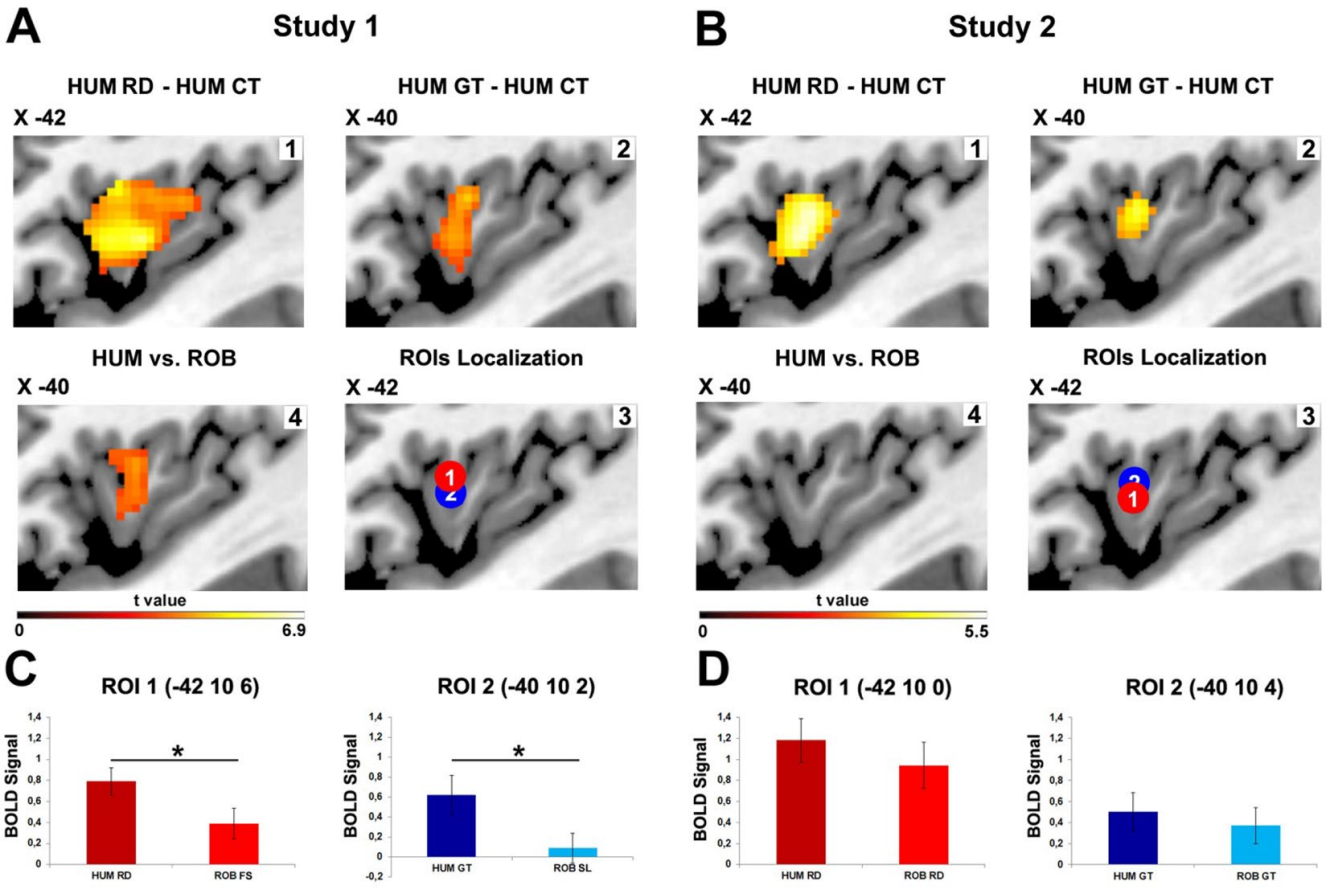
Insular activations during the processing of actions performed by human actors and iCub robot. Parasagittal sections showing the activation of the left insula resulting from the contrasts *HUM RD versus HUM CT* and *HUM GT versus HUM CT* obtained in Study 1 (**A**, panels 1–2) and Study 2 (**B**, panels 1–2) (P_FWE_ < 0.05 cluster level). Around the local maxima of these activation foci, two ROIs were build [Study 1, panel A3 (ROI 1: x = -42, y = 10, z = 6; ROI 2: x = -40, y = 10, z = 2); Study 2, panel B3 (ROI1: x = -42, y = 10, z = 0; ROI2: x = -40, y = 10, z = 4)]. The panels A4 and B4 show the insular activation produced by the contrast *human versus robot* regardless of the type of vitality form conveyed. BOLD Signal changes extracted in the two ROIs located in the dorso-central insula in Study 1 (**C**) and Study 2 (**D**). The bar graphs indicate the comparisons between *human* and *robot* in the two experimental conditions. Asterisk indicates the significant difference (*p* < 0.05).

The most important results with regards to assessing the role of vitality forms in the modulation of brain activity associated with human actions are derived from the contrasts *human rude actions (HUM RD) versus human jerky actions (HUM CT)* and *human gentle actions (HUM GT) versus human jerky actions (HUM CT)*. Results of both contrasts revealed the activation of the left dorso-central insula (Fig. 2A, panels 1–2). In contrast, the observation of *robotic actions generated with low and high velocities* relative to controls *[robotic fast actions (ROB FS) vs. robotic jerky actions (ROB CT); robotic slow actions (ROB SL vs. robotic jerky actions (ROB CT)]* did not produce the activation of the dorso-central insula. Additionally, the contrast *robotic fast actions versus robotic jerky actions* produced the activation of the inferior frontal gyrus, prefrontal gyrus and superior temporal gyrus in the right hemisphere.

#### Contrasts between human and iCub robot

The most important result for assessing possible differences between the perception of human and robotic actions are derived from the direct contrast between *human versus robot* [human (*rude actions* + *gentle actions*)—robot (*fast actions* + *slow actions*)]. Specifically, this contrast revealed activation of the left dorso-central insula (Fig. 2A, panel 4). Conversely, the opposite contrast (robot vs. human) did not produce any significant activation. It is important to note that the activation of the dorso-central insula was also found in the direct contrast between *human gentle actions* versus *robotic slow actions*.

### fMRI study 2

**Figure 3:**
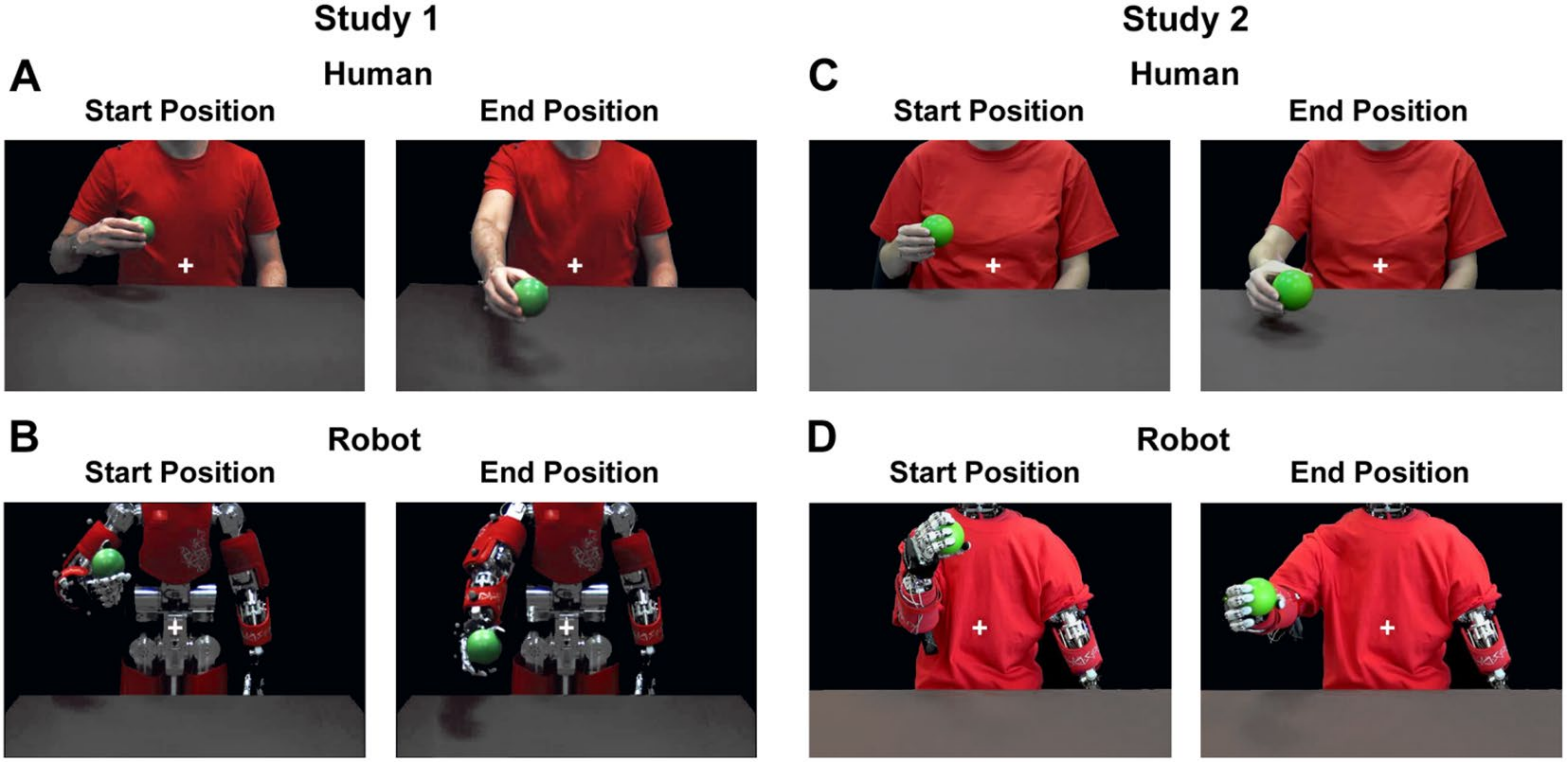
Example of video-clips observed by participants during the two experiments. Study 1: the images show the human actor and the iCub robot at start (**A**, **B**, left panels) and end positions (**A**, **B**, right panels). Study 2: the images show the human actress and the iCub robot dressed in a red shirt at start (**C**, **D**, left panels) and end positions (**C**, **D**, right panels). For each action, the initial and final positions were fixed. In the central part of the screen a white cross indicated the focus point for the duration of videoclips.

**Figure 4:**
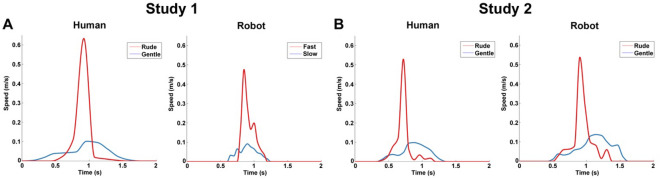
Physical properties of the video stimuli. Graphs depict the profiles of 2D mean velocity perceived by participants during the observation of video-clips showing gentle and rude actions performed by human actors and iCub robot in Study 1 (**A**) and Study 2 (**B**).

Sixteen healthy right-handed volunteers (5 females and 11 males, mean age = 25, SD = 3) participated in the second fMRI studies. The stimuli presented in this fMRI study were very similar to those presented in the first study [2 conditions: *human* and *robot*; 1 action performed with 5 objects with vitality forms (gentle and rude) or without vitality form (jerky action)] (Fig. 3C). Most importantly, in the *robot condition*, participants were presented with video-clips showing the iCub robot executing and exactly replicating of the actions generated by humans. In this way, the robot’s movements implicitly contain the kinematic parameters characterizing human vitality forms (peak velocity, velocity profile: Fig. 4B). As in Study 1, during the presentation of the stimuli participants were required to focus on a white cross in the center of the screen and pay attention to the way in which actions were performed.

#### Main effect of actions performed by human and iCub robot

Results indicate that the observation of actions performed by humans either gently or rudely produced a common activation of the occipital lobe, bilateral inferior parietal lobule, bilateral premotor cortex and inferior frontal gyrus extending to the insula (Fig. 1C, left panel). A very similar activation pattern was observed for the *robot condition* with a large extension of the frontal areas including the insular cortex (Fig. 1D, left panel, see Table 2 for coordinates). Furthermore, the observation of video-clips showing jerky actions (control stimuli) performed by both human and robot, produced similar activation patterns, though more extensively in the left inferior frontal gyrus and in the right hemisphere for the *robot condition* (Fig. 1C,D, right panels).

**Table 2:** Cerebral activity obtained in Study 2 during the observation of (**A**) *human actions versus baseline* (global null conjunction of *gentle* and *rude* conditions); (**B**) *robotic actions versus baseline*. Local maxima, as shown in Fig. 1C,D, are given in MNI standard brain coordinates, significant threshold is set at *P*_FWE_ < 0.05 (cluster-level).

Anatomical region	Left hemisphere				Right hemisphere			
	x	y	z	Z-score	x	y	z	Z-score
**(A) Human actions versus baseline**								
Inferior occipital gyrus	− 48	− 70	− 4	Inf	42	− 68	− 16	Inf
Superior occipital gyrus					20	− 88	6	Inf
Middle occipital gyrus	44	− 76	− 2	Inf				
Lingual gyrus					12	− 90	− 6	Inf
Fusiform gyrus					32	− 72	− 14	Inf
Inferior Temporal Gyrus					58	− 60	− 4	Inf
Middle temporal gyrus	− 48	− 64	8	Inf	50	− 64	− 2	Inf
Inferior parietal lobule	− 42	− 48	48	5.53				
Precentral gyrus	− 42	− 4	46	6.22	42	2	44	Inf
Middle cingulate cortex					8	16	44	5.20
Posterior-medial frontal gyrus					6	6	70	6.43
Insula	− 42	12	0	4.49				
Hippocampus	− 22	− 28	− 6	7.25				
Thalamus					20	− 28	− 2	Inf
Cerebellum	− 10	− 76	− 40	6.90	22	− 76	− 20	Inf
**(B) Robotic actions versus baseline**								
Inferior occipital gyrus					44	− 76	− 2	Inf
Calcarine gyrus	− 4	− 84	− 12	Inf	14	− 90	2	Inf
Lingual gyrus	− 6	− 78	− 4	Inf	12	− 90	− 6	Inf
Precuneus	− 6	− 40	70	4.99	6	− 52	68	4.10
Fusiform gyrus					28	− 70	− 14	Inf
Middle temporal gyrus					50	− 62	0	Inf
Paracentral lobule	− 12	− 32	72	3.52	10	− 30	68	4.81
Inferior parietal lobule	− 44	46	46	5.17				
Precentral gyrus	− 48	2	52	5.75	42	2	44	7.80
Inferior frontal gyrus					40	28	− 4	4.44
Posterior-medial frontal gyrus	− 6	10	50	5.43	4	8	70	5.57
Insula	− 42	12	0	3.81				
Hippocampus					28	− 24	− 8	Inf
Thalamus					22	− 26	− 4	Inf
Cerebellum					22	− 76	− 20	Inf

#### Contrasts between vitality forms and control stimuli

The direct contrast between human actions performed with *vitality forms* (gentle or rude) and the *control* condition (jerky actions) revealed the activation of the left dorso-central insula (*HUM RD vs. HUM CT; HUM GT vs. HUM CT*; Fig. 2B, panels 1 and 2). On the contrary, the contrast between the observation of robotic actions and the control condition did not produce any activation [*robotic rude actions (ROB RD) vs. robotic jerky actions (ROB CT)*; *robotic gentle actions (ROB GT) vs. robotic jerky actions (ROB CT*)]. The lack of insular activity may have been due to the extensive activation of the left inferior frontal gyrus during the observation of *robotic jerky actions* (Fig. 1D, right panel).

#### Contrasts between human and iCub robot

Unlike the results of Study 1, the direct contrast between the observation of human actions and robotic actions [human (*rude actions* + *gentle actions*) – robot (*rude actions* + *gentle actions*)] did not reveal any significant activation (Fig. 2B, panel 4). Furthermore, no significant activation was found in the contrast *human rude actions versus robotic rude actions* and in the contrast *human gentle actions versus robotic gentle actions.*

#### Testing for the vitality effect in the dorso-central insula: region-of-interest analysis

In both Studies, in order to examine the BOLD signal change of the insular cortex during the observation of human and robotics actions and quantify possible differences (Study 1: *HUM RD vs. ROB FS, HUM GT vs. ROB SL*; Study 2: *HUM RD vs. ROB RD, HUM GT vs. ROB GT*), two regions of interest (ROIs) were defined. These ROIs were created using the functional maps of the contrasts analyses obtained in Study 1 and Study 2. In particular, ROI 1 was defined by centering the sphere (radius = 3 mm) around the local maxima of activation resulting from the contrast *HUM RD versus HUM CT* [ROI 1 coordinates: x = − 42, y = 10, z = 6 (Study 1); x = − 42 , y = 10, z = 0 (Study 2)], whereas ROI 2 was created around the maxima of activation resulting from the contrast HUM GT versus HUM CT [ROI 2 coordinates: x = − 40, y = 10, z = 2 (Study 1); x = − 40, y = 10, z = 4 (Study 2); Fig. 2A3–B3]. Then, using the SPM Rex Toolbox (https://web.mit.edu/swg/rex), in both ROIs, for each participant, the BOLD signal change was extracted and recorded relative to the observation of human and robotic actions performed in different conditions (Study 1: *HUM RD, HUM GT, ROB FS, ROB SL;* Study 2: *HUM RD, HUM GT, ROB RD, ROB GT*). Considering the BOLD signal extracted from ROIs of Study 1, *t*-tests revealed significant differences between the human and iCub robot during the observation of rude (ROI 1, Fig. 2C, left panel) and gentle actions (ROI 2, Fig. 2C, right panel; *p* < 0.05). In contrast, *t*-tests relative to the BOLD signal extracted in ROIs of Study 2 did not reveal any difference between human and iCub robot (Fig. 2 D; *p* > 0.05).

## Discussion

During social interactions, according to positive or negative mood, the agent may perform actions with different forms communicating his/her internal state. The observation of these forms of action allows the receiver to understand the internal state of the agent (“how she/he feels”) and consequently to prepare an adequate motor response. Previous fMRI data have shown that the dorso-central sector of insula is a key region involved in the perception and expression of these aspects of social communication named vitality forms (Daniel Stern)^10–12,24^. In this study, two fMRI experiments were conducted to investigate whether the observation of actions executed by a humanoid robot (iCub) could elicit activation of the dorso-central insula as well as investigate which kinematic parameters are responsible for the activation. In the first study participants were required to pay attention to video-clips showing an actor performing actions with different vitality forms (gentle and rude) and robotic actions generated with low and high velocities which were designed to be reminiscent of the gentle and rude action vitality forms performed by the actor. In agreement with previous data, observation of human actions produced a bilateral activation of the parieto-frontal circuit known to be involved in the encoding of action execution and recognition^25,26^. The same circuit was also activated during the observation of robotic actions. Most interestingly, results from this first experiment showed that, besides the common activation of the parieto-frontal circuit, only observations of human actions produced activation of the dorso-central insula. Furthermore, the ROI analysis carried out in this insular sector showed a significant difference of the BOLD signal between the observation of human actions and robotic actions in both rude and gentle conditions. Taken together, these findings indicated that observations of iCub robot generating actions with slow and high velocities produced activation of the parieto-frontal circuit but not of the insula suggesting that the observer understood the action goal (passing the object, “what”) but not the way in which the action was performed i.e. its vitality form (“how”). The specific activation of the insula only for observations of human actions could be due to the physical properties of the stimuli which, in the case of the “biological motion” model used to generate iCub movements, did not include vitality. Indeed, the kinematic parameters (e.g. peak velocity, length trajectory) of human actions differed from those generated by iCub robot. To test this possibility, a second fMRI study was conducted to investigate whether the observation of robotic actions generated by producing an exact replica of the human actions (i.e. same kinematic parameters including peak velocity) could elicit activation of the insula. In this study, as was the case for the human condition, the robot wore a red shirt to emphasize arm actions. Results of Study 2 confirmed this hypothesis, showing that observation of robotic actions endowed with human vitality forms induced a BOLD signal increase in the dorso-central insula. In particular, the ROI analysis carried out in the dorso-central insula did not reveal any significant difference of the BOLD signal between the observation of human and robotic actions in both gentle and rude conditions. Most interestingly, conversely to the first study, the main contrast for assessing possible differences between the perception of human and robotic actions (*human actions vs. robotic actions*) revealed no differences in activation of the insula. These data suggest that the dorso-central insula was involved in the processing of both human actions and robotic actions conveying human vitality forms. One could object that this insular activity, besides corresponding to the recognition of vitality forms generated by the iCub robot, may also reflect a possible effect of the shirt that the robot wore. The choice of “dressing” the iCub robot was made to avoid possible confounding effects that may affect participant attention (e.g. rotation, glittering, etc.), emphasizing the arm actions as occurred in the human condition. Although this hypothesis cannot be completely excluded, the observation of the iCub robot wearing the shirt and generating actions with a jerky movement (control condition) did not produce the activation of the dorso-central insula *(robot jerky actions vs. baseline)*.

Taken together, data obtained from Study 1 and Study 2 confirm the role of the dorso-central insula in the processing of vitality forms indicating that this brain area is also involved in the processing of robotic actions conveying human vitality forms. Furthermore, these two fMRI studies provide evidence that the kinematic parameters of human actions play a fundamental role in triggering the activation of the dorso-central insula. Notably, insula activity increased during the observation of a humanoid robot performing actions with biological kinematic parameters characterizing human vitality forms (peak velocity, velocity profile). Additionally, although the two fMRI studies were conducted with two different groups of participants, the activation foci obtained during the observation of human vitality forms (*human rude actions vs. human control actions*; *human gentle actions vs. human control actions*) were located in the same insular sector (Fig. 2A,B, panels 1 and 2).

It is not completely clear how visual information reaches the dorso-central insula, during the observation of actions performed by others, allowing for the recognition of vitality forms. Previous anatomical data obtained from monkeys showed that the dorso-central insula receives connections from the anterior part of the superior temporal sulcus^27,28^, where neurons are located that respond to complex visual stimuli, amongst which are several types of arm movements. Similarly, in humans, Almashaikhi and colleagues^29^ showed that electrical stimulation of the dorso-central insula (middle and posterior short gyri) in patients with drug-resistant epilepsy, determines evoked potentials in the temporal areas encoding biological visual stimuli, such as the observation of hand and arm actions. This cortical network represents the main pathway through which visual input may reach the dorso-central insula. After the encoding of vitality forms, it is very likely that the insula may transform this information in a motor domain allowing the observer to prepare an adequate motor response. This hypothesis is corroborated by anatomical studies conducted with monkeys showing that the central part of the insula is connected with the parieto-frontal areas (the inferior parietal lobule; the ventral premotor cortex, and the prefrontal area 46) which have a fundamental role in the control of voluntary hand/arm movements^30–32^. These findings are also in agreement with a tractography study (DTI) conducted with both humans and monkeys (Di Cesare and colleagues^33^) showing that the dorso-central insula is connected with the parieto-frontal circuit in both species. These anatomical connections confirm the involvement of the dorso-central insula in the cerebral networks for generating hand actions suggesting its possible role in the modulation of action. In line with this hypothesis, previous studies have indicated that the dorso-central insula is activated not only during the observation of vitality forms but also during their execution. The overlap of execution and perception of action conveying vitality forms suggests the existence of a mirror mechanism for action vitality forms in the dorso-central insula. This mirror mechanism is different from that located in the parietal and frontal areas specific for the understanding of action goals, and is located in the insula which might allow one to express their own mood/attitude and to understand those of others.

In conclusion, three particular findings from this study add to the existing data on the observation of robotic actions and shed new light on the functioning of the dorso-central insula: first, the observation of robotic actions does not interfere per se with activation of the insular cortex. Second, kinematic features (e.g. peak velocity) of an observed action appear to represent an important cue essential for triggering insula activity. Third, observing a humanoid robot performing actions generated with human kinematic parameters such as peak velocity, as well as velocity profile, produce activation of the dorso-central insula.

This study represents a preliminary step in understanding how to endow robots with gentle and rude vitality forms, as well as the neural correlates involved in the processing of these robotic vitality forms. Furthermore, it highlights how programming robot movements in a precise and repetitive way could allow for the investigation of the contributions of different kinematic parameters to perception of actions. Results and methodology from this study may have implications for other research fields such as robotics and private sectors. In fact, robots in the future are expected to interact with humans in several scenarios including as a companion^34–36^, or assistant^37^. These new applications will require robots to be calm, efficient, legible and entertaining. To succeed in these new applications, robots need to efficiently communicate with humans in their environment^38–40^. It is plausible that, in future, vitality forms could become a fundamental tool for humanoid robots and virtual avatars allowing them to convey the internal states that characterize human communication. In this way, robots will appear more authentic and comfortable to interact with, promoting interactions with humans.

## Materials and methods

### Subjects

Two different groups of sixteen healthy right-handed participants (group 1: six females and ten males, mean age = 26.5 years, SD = 3.8; group 2: five females and eleven males, mean age = 25.7 years, SD = 3.4) took part in Study 1 and Study 2 respectively. The choice to collect 16 subject in the current study is based on results provided by a power analysis carried out on previous data relative to a similar fMRI study^12^. Results of power analysis indicated that, in order to obtain a medium effect in the dorso-central insula due to the observation of human actions conveying vitality forms, it is essential to collect a sample consisting of at least 15 participants [(partial eta square = 0.21, α = 0.05, β = 0.95 and non sphericity correction 0.7 (ε)]. All participants had normal or corrected-to-normal vision. Informed consent was obtained from all participants and the experiment was approved by the ethics committee of the University of Parma (UNIPRMR750v1) in accordance with the Declaration of Helsinki.

### Experimental design, stimuli and tasks

Both Study 1 and Study 2 consisted of three experimental runs. Each run was organized in a blocked design based on a 2 × 3 factorial design with the *agent* (*human* and *robot*) and the *action forms* [*gentle actions, rude actions (vitality forms condition) and jerky actions (control condition)*] as factors.

In Study 1, participants were shown video clips of a male actor offering different objects (apple, orange, packet of crackers, bottle, ball) with gentle or rude vitality forms, or without any vitality form (i.e. jerky actions; *control condition*) (Fig. 3A). Specifically, control stimuli were obtained by presenting one static frame of the action every 400 ms (5 frames in total from the beginning to the end of the action). The aim of the control stimuli was to allow participants to understand the action goal without conveying any vitality form information. Additional video-clips showed a humanoid robot (iCub) generating the same actions (offering different objects) at one of two velocities (high or low) which approximate the gentle and rude vitality forms expressed by the human actor (Fig. 3B). As a control, participants were presented with video-clips showing the iCub robot generating the same actions with a jerky movement. The iCub robot generated actions in accordance with the 2/3 power law which characterizes biological motion^41^. Subsequently, in order to obtain a similar peak velocity between the kinematic curves of actions performed by the human actor and those generated by the iCub robot, the frame rate of video-clips was adjusted accordingly (Fig. 4A). For all video-clips, the face area of both the human actor and iCub robot was omitted to avoid possible confounding effects due to attention being paid to facial expression. All video-clips were recorded using a high definition camera (Panasonic HC X900) fixed at a 180° angle with respect to the human actor and the iCub robot (i.e., providing an allocentric point of view).

In Study 2, participants were presented with very similar video stimuli as those presented in Study 1 [2 agents: *human* and *robot*; 3 action types: *gentle actions, rude actions (vitality forms condition) and jerky actions (control condition)*] differing on three points: 1) the human agent was a female actress; 2) the iCub robot was able to execute the exact kinematic replica of the actions performed by the human actress (i.e. with the same velocity profiles); and 3) the iCub robot wore a red shirt to emphasize the arm actions as occurred for the human actor (Fig. 3D).

The choice of a female actress was supported by the necessity of matching the anthropometric characteristics of the iCub robot with those of the human agent in order to match the kinematic parameters (velocity profile, length trajectory) of action. For this purpose, the kinematic parameters regarding gentle and rude actions performed by the actress were recorded by the OptoTrack system and remapped into the kinematic model of the iCub robot refining the generation of its actions.

All actions performed in Study 1 and Study 2 were also filmed by a high definition camera (Panasonic HC X900) in order to obtain video stimuli to present in the two fMRI studies.

**Figure 5:**
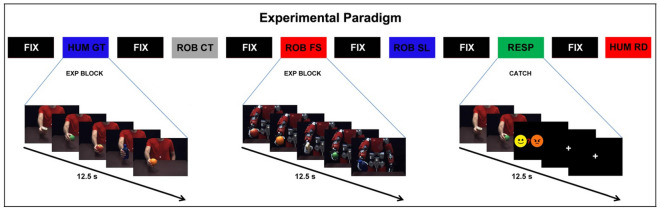
Graph shows the experimental paradigm adopted in the two fMRI studies. A block design was used in both experiments. Video stimuli were presented in blocks of five consecutive stimuli (duration 12.5 s) of the same condition (*human gentle actions*, HUM GT; *human rude actions*, HUM RD; *human jerky actions*, HUM CT; *robotic slow actions*, ROB SL; *robotic fast actions*, ROB FS; *robotic jerky actions*, ROB CT) followed by a rest period lasting 12.5 s (FIX). In 10% of cases, catch trial blocks (Resp) were intermixed with experimental blocks, in which participants had to indicate the last observed vitality form (gentle, rude, neutral).

During the experiment, participants were required to fixate on a white cross in the center of the screen and to focus on the vitality forms of the action. At the beginning of each block, an instruction panel stating “observe” reminded participants to pay attention to the action. In 10% of cases, participants were required to indicate the last perceived vitality form by pressing a button on a response box placed inside the scanner (Fig. 5).

### Kinematic recording and physical properties of stimuli

#### 3D stimuli motion

In Study 1, a VICON Motion Capture System (Vicon OMG, UK MX2 model, sampling frequency: 100 Hz.) was used to record the kinematic features of actions performed by both the male actor and the robot. In particular, six infrared cameras recorded the 3D position (at regular time intervals) of 5 markers placed in different positions of the right arm: one on the thumb, one on the index finger, one on the wrist, one on the elbow and one on the shoulder. In all videos, the actor and the robot performed the actions starting from the same initial position and reaching the same final position (Fig. 3A). During execution, the natural and ecological expression of human actor was preserved as much as possible avoiding excessive artificial manipulation of kinematic variables.

In Study 2, the kinematic features of the actions performed by both the female actress and the robot were recorded from the same marker positions as described above, but using a different system: the NDI OptoTrack (sampling frequency: 200 Hz). The choice of this new recording system was supported by the presence of active markers. These specific markers helped to avoid possible confounding effects caused by the reflective metallic parts of the robot.

The Two-Thirds (2/3) Power Law^41^ is a typical trait of human motion, linking speed to the curvature of an elliptical movement. A module was designed giving the iCub robot the ability to execute curve arm movements compliant with this law^42^, taking advantage of the existing Cartesian controller of the iCub^43^. Given some precise parameters that define a trajectory in 3D space, the module can generate a smooth, human-like movement, which is then executed by the robot. Particular attention was paid to the generation of biological motion for the robot because, as has been previously demonstrated, such motions are decisive in drawing out motor resonance and automatic imitation during interactions^44,45^.

### 2D perceived velocity of stimuli

After video recording, the 2D kinematic profiles of all actions presented in the video-clips were analyzed using the Tracker software. For this purpose, a specific point of the agent’s hand (human or robot) corresponding to the index finger, was marked for all video clips. In particular, for each video frame, the position of the index finger was tracked in the space from the beginning to the end of action. For all actions, using both X and Y values of each tracked point, the module of velocity $\left( {\left| {\text{v}} \right| = \sqrt {{\text{v}}_{{\text{x}}}^{{2}} + {\text{v}}_{{\text{y}}}^{{2}} } } \right)$ for gentle and rude actions was calculated and averaged. Figure 4 shows the kinematic profiles of human and robotic actions presented to participants in Study 1 and Study 2 (perceived velocity).

### Experimental paradigm

Participants both studies were laid in the GE MR750 scanner in a dimly lit environment. The stimuli were presented via the VisuaSTIM system with a 30 dB noise-attenuating headset with 40 Hz to 40 kHz frequency response and with a 500.000 pixels × 0.25 square inch resolution with horizontal eye field of 30°. The transmission of the digital signal to the scanner occurred via optic fiber. E-Prime 2 Professional software was used (Psychology Software Tools, Inc., Pittsburgh, USA, https://www.pstnet.com) in order to present video stimuli and record participants’ answers. Video stimuli were presented in blocks of five consecutive stimuli of the same condition each lasting 2.5 s (conditions: *human gentle actions, human rude actions; human jerky actions; robotic slow actions* (Study 1) *or robotic gentle actions* (Study 2)*; robotic fast actions (*Study 1*) or robotic rude actions (*Study 2*); robotic jerky actions*). An inter block period of 12.5 s without video stimuli was present between two consecutive blocks. In 10% of cases the inter block period catch trials were randomly presented and participants were required to indicate the last vitality form observed by pressing a button on a response box placed inside the scanner (Fig. 5). Study 1 and Study 2 were both composed of three functional runs comprising a total of nine blocks (45 single trials) for each condition, presented in a randomized order. Each functional run lasted approximately 10 min.

### fMRI data acquisition

Imaging data were collected on a 3 T Discovery MR750 GE scanner equipped with an eight-channel receiver head coil. Functional images were acquired using a gradient EPI sequence with a TR of 2500 ms, TE of 30 ms, flip angle of 90°, parallel imaging acceleration factor of 2, 205 × 205 mm^2^ field of view, voxel size of 2.5 × 2.5 × 3 mm^3^. The scanning sequence comprised 224 ascending sequential volumes composed by 40 axially slices. Additionally, a high resolution T1-weighted structural image (1 × 1 × 1 mm^3^) was acquired with a TR of 8100 ms, TE of 3.2 ms, flip angle of 12° for each participant.

### Statistical analyses

Data analyses for both Study 1 and Study 2 were performed using SPM12 (Wellcome Trust Center for Neuroimaging, London, UK). The first three volumes of each run were discarded to allow for T1 equilibration effects. For each participant, functional volumes were first slice-timing corrected, realigned to the mean volume and unwarped for between-scan motion correction. Subsequently, the T1-weighted image was resampled into functional image space before segmentation into gray, white and cerebrospinal fluid and normalization to the Montreal Neurological Institute (MNI) space, according to SPM12 preprocessing pipeline. Finally, spatial transformations derived from segmentation step were then applied to the realigned EPIs for normalization to MNI space with a voxel size of 2 × 2 × 2 mm. At the end of preprocessing, all functional normalized volumes were spatially smoothed with a 6 mm full-width half maximum isotropic Gaussian kernel. For all subjects, head motion was carefully checked and no participant met the exclusion criteria of 3 mm mean displacement. Data were analyzed using a random-effects model^46^, implemented in a two-level procedure. At the first level (single-subject analysis), the BOLD signal was modeled using a general linear model (GLM) comprising the onsets and durations of each block condition for each functional run. In the first Study, the GLM model consisted of eight regressors: *human rude actions (HUM RD)*, *human gentle actions (HUM GT)*, *human jerky actions (HUM CT)*, *robot*ic *fast actions (ROB FS)*, *robotic slow actions (ROB SL)*, *robotic jerky actions (ROB CT)*, *instruction* and *response*. Stimuli were presented in blocks of five consecutive video-clips of the same condition. Each block was modeled as a whole event lasting 12.5 s. The blocks presenting catch trials intermixed with the experimental blocks were modeled as events lasting 12.5 s (Fig. 5).

In the second-level analysis (group analysis), contrast images of the first level were entered into a flexible factorial model for each participant^47^. This model consisted of six regressors (*HUM RD*, *HUM GT*, *HUM CT*, *ROB FS*, *ROB SL*, *ROB CT*) and considered the activation patterns resulting from contrasts between conditions (*HUM RD vs. HUM CT; HUM GT vs. HUM CT; ROB FS vs. ROB CT; ROB SL vs. ROB CT*) and agents (*HUM vs. ROB; HUM RD vs. ROB FS; HUM GT vs. ROB SL*).

In the second Study, the GLM model consisted of eight regressors at the first level: *HUM RD*, *HUM GT*, *HUM CT*, *ROB RD*, *ROB GT*, *ROB CT*, *instruction* and *response*. In the second-level analysis, for each participant, the contrast images of the first level were entered into a flexible factorial model^47^. This model consisted of six regressors (*HUM RD*, *HUM GT*, *HUM CT*, *ROB RD*, *ROB GT*, *ROB CT*) and considered the activation patterns resulting from contrasts between conditions (*HUM RD vs. HUM CT; HUM GT vs. HUM CT; ROB RD vs. ROB CT; ROB GT vs. ROB CT*) and agents (*HUM vs. ROB; HUM RD vs. ROB RD; HUM GT vs. ROB GT*).

In both studies, the location of the activation foci was determined in the stereotaxic space of the MNI coordinates system. All significant clusters were identified using an a priori voxel-wise FWE-corrected threshold of *p* < 0.05 (cluster level).
